# Study on the Macro and Micromechanics Tensile Strength Properties of Orange Tree Pruning Fiber as Sustainable Reinforcement on Bio-Polyethylene Compared to Oil-Derived Polymers and Its Composites

**DOI:** 10.3390/polym12102206

**Published:** 2020-09-25

**Authors:** Francesc X Espinach, Eduardo Espinosa, Rafel Reixach, Alejandro Rodríguez, Pere Mutjé, Quim Tarrés

**Affiliations:** 1 Design, Development and Product Innovation, Dept. of Organization, Business, University of Girona, 17003 Girona, Spain; 2 Chemical Engineering Department, Bioagres Group, Faculty of Science, Universidad de Córdoba, Campus of Rabanales, 14014 Córdoba, Spain; a02esvie@uco.es (E.E.); q42ropaa@uco.es (A.R.); 3 Laboratori d’Enginyeria Paperera i Materials Polímers (LEPAMAP Research Group), Universitat de Girona, Campus Montilivi, C.P., 17003 Girona, Spain; rafel.reixach@udg.edu (R.R.); pere.mutje@udg.edu (P.M.); joaquimagusti.tarres@udg.edu (Q.T.); 4 Càtedra de Processos Industrials Sostenibles, Universitat de Girona, Campus Montilivi, C.P., 17003 Girona, Spain

**Keywords:** bio-based, composites, natural fibers, mechanical properties

## Abstract

Agroforestry creates value but also a huge amount of waste outside its value chain. Tree pruning is an example of such a low value waste, that is typically discarded or incinerated in the fields or used to recover energy. Nonetheless, tree prunings are rich in wood fibers that can be used as polymer reinforcement. Although there are some bio-based polymers, the majority of industries use oil-based ones. The election of the materials is usually based on a ratio between properties and cost. Bio-based polymers are more expensive than oil-based ones. This work shows how a bio-polyethylene matrix can be reinforced with fibers from orange tree prunings to obtain materials with notable tensile properties. These bio-based materials can show a balanced cost due to the use of a cheap reinforcement with an expensive matrix. The matrix used showed a tensile strength of 18.65 MPa, which reached 42.54 MPa after the addition of 50 wt.% of reinforcement. The obtained values allow the use of the studied composite to replace polypropylene and some of its composites under tensile loads.

## 1. Introduction

Agricultural activity, indispensable for human beings, needs to be restructured from many points of view, one of which is the economy. Nowadays, agriculture benefits from government subsidies that try to alleviate, in some way, the low price farmers receive for their products. Farmers use materials and human resources for the cultivation and development not only of fruits and seeds but also for the growth of the plant structure that shelters them, which once harvested become agroforestry waste. The widespread use of these wastes hardly succeeds in modifying the economic balance of the activity. A sustainable economy or bioeconomy, to be so, must use renewable resources and be, at the same time, circular [1], integrating each of the elements that constitute the value chain of production and obtain environmental, social, and economic benefits.

As mentioned above, agricultural activity generates enormous amounts of waste. These wastes are mainly composed of cellulose, lignin, and hemicellulose, and are known as vegetable biomass or lignocellulosic biomass. There are multiple applications for each of these constituent fractions: active carbons [2], composites [3], levulinic acid production [4], thermal insulating materials [5], viscose [6], materials for the treatment of wastewater [7], edible coatings [8], furfural and hydroxymethylfurfural production [9], the formation of thermoplastics and flexible films [10], obtaining prebiotics applicable in the food industry [11], acid dyes absorbents [12], food additives and nutraceuticals [13], energy, fuels, synthesis gas, macromolecules, and aromatic compounds [14,15,16] and composites [17,18], among others.

Orange cultivation is widely spread all over the world. According to FAO data, more than 75 million tons were grown in 2018, with Brazil, the United States, China, India, Mexico, and Spain representing about 60% of this production [19]. In Spain, oranges are produced mainly in the Guadalquivir Valley and the Mediterranean region. Spanish orange production in 2018 amounted to more than 3.5 million tons [20].

The pruning of the orange tree, which is carried out annually, produces large quantities of lignocellulosic waste that is used for purposes that are not economically relevant: agricultural amendments, the formation of pellets and burning to recover energy when it is not burned directly in the field for disposal. A reasonable ratio between residue/fruit can be 1/0.8 [20], so taking into account the annual production, huge amounts of waste are generated each year.

Orange tree pruning can be exploited following the 12 principles of green chemistry, especially principle 7 (use of renewable raw materials). Then, to fulfill principles 5 (use of safer solvents and auxiliaries) and 6 (energy efficiency) it must be processed in the simplest way possible. It can be extremely interesting and useful to combine these three principles to make better use of these wastes. Moreover, it is better to prevent waste than to treat or clean up waste after it has been formed. In this sense, obtaining high yield pulps will help us to generate the minimum sub-waste in the residue valorization.

In addition, the 12 Principles of Green Chemical Engineering, enunciated by Anastas and Zimmerman [21], like the previous 12 principles, provide a framework for the activity of scientists and engineers to converge in the design of new materials, products, processes, and systems that are beneficial to health and the environment. The 4th principle refers to design with the resource maximization as a goal (products, processes, and systems should be designed to maximize mass, energy, space, and time efficiency). Furthermore, principle 11 calls for the design of recyclable products as a contribution to the circular economy. Products, processes, and systems should be designed for performance in a commercial “afterlife”.

Europe in general and Spain in particular consider that it is necessary to boost the economic development of rural areas. Thus, the generation of qualified jobs in sectors related to the agricultural activity will favor an increase in the standard of living, avoiding the depopulation of rural areas. To help achieve this objective, it is essential to increase the competitiveness of agriculture by developing models for managing natural resources that allow for the balanced development of the rural world. This work proposes initiatives that can be developed in a rural environment by adding value to agri-food waste.

Most plastics used today come from the exploitation of fossil resources and are therefore not renewable nor biodegradable. These include polyolefins, the most frequently used polymeric materials, which are also reinforced, mostly with glass fiber (GF), for use in applications where the matrix alone is not applicable. The need to reduce or eliminate dependence on fossil resources and to produce environmentally friendly materials has been identified as one of the key issues for sustainable development [22,23,24,25,26,27,28]. In this regard, two main types of environmentally sustainable plastic materials have emerged; biodegradable bio-based materials and recyclable bio-based materials [24,29,30,31,32]. One of the main objectives of research into these greener materials is to achieve properties similar to those of current commercial materials based on non-renewable resources. In this sense, biopolyethylene, produced from sugarcane [33], has the same properties as polyethylene obtained from petroleum. On the other hand, it is possible to produce fully bio-based composite materials, based on bioplastics, to obtain the mechanical properties required for a wide range of applications [34,35]. Biocomposites based on biopolyethylene and natural fibers are a good example of bio-based and recyclable materials [36]. Nowadays, one of the main drawbacks of biopolyethylene is its price, which is not competitive in comparison with oil-based polyethylene [37]. The use of natural fibers as fillers can decrease the cost of the biocomposites and making them more competitive. However, there are some factors to consider when natural fibers are incorporated into polymeric matrices. Among them, the degradation temperature of the natural fibers, around 220 °C [38], and the fiber-matrix compatibility [39]. In the case of biopolyethylene composite materials, the degradation temperature of the fibers does not have an important impact since this matrix can be processed below 200 °C. However, the chemical structure of biopolyethylene, which is identical to polyethylene from fossil resources, is hydrophobic and requires compatibility with natural hydrophilic fibers. Compatibility between natural fibers and the plastic matrix can be achieved by using coupling agents such as maleated polyethylene (MAPE) [40,41]. However, the chemical composition of the fiber surface is also a key factor [42]. The chemical composition of the raw material as well as the treatments applied to obtain the fibers will lead to fiber-matrix interfaces particular to each case [43,44].

This work has focused on obtaining biocomposites with competitive mechanical properties based on bio-polyethylene reinforced with orange tree pruning fibers. This includes the compatibilization of the phases to obtain strong fiber-matrix interfaces. For this purpose, composite materials with MAPE contents ranging from 0 to 10 *w/w* % were produced and characterized, to study the behavior of the traction resistance of such composites against MAPE content to obtain the MAPE dosage that ensures the highest traction resistance. Based on this MAPE content, the tensile strength of composites with 20 to 50 *w/w* % of orange pruning fibers was studied. A micromechanical analysis of the tensile strength was carried out to further understand the behavior of the fiber-matrix interface, obtaining its shear strength and the intrinsic tensile strength of the reinforcements.

## 2. Materials and Methods

### 2.1. Materials

Orange tree prunings (*Citrus sinensis*) were supplied by cooperatives in Palma del Río (Córdoba, Spain). The original prunings included leaves and branches of different diameters. To achieve homogeneous materials, any stalks with diameters lower than 10 mm were discarded.

The matrix was a biopolyethylene (bioPE) supplied by Braskem (Sao Paulo, Brazil). This polymer is distributed under the commercial name SHA7260. The Bio-PE is injection-grade and has a molecular weight of 61.9 g/mol.

A polyethylene functionalized with maleic anhydride (MAPE) was used as a coupling agent. This MAPE was supplied by Eastman Chemical Products (San Roque, Spain) and is commercialized under the tradename Fusabond MB100D. For comparison purposes, Rigidex HD5226EA high-density polyethylene (HDPE) supplied by INEOS Polyolefins (Barcelona, Spain), and glass fibers (GF) by Saint-Gobain Vetrotex (Gorlice, Poland) were used to produce composites at 20 and 30% with MAPE.

Decahydronaphtalene (decalin) provided by Fisher Scientific (Barcelona, Spain) was used to dissolve the matrix and recover the fibers from the composites. This reagent has a 190 °C boiling point and a purity of 97%.

### 2.2. Methods

#### 2.2.1. Chemical Characterization of the Orange Tree Pruning

Orange tree prunings were characterized as follows: holocellulose α-cellulose, lignin, ash, and ethanol-benzene extractives according to TAPPI standards T-9m54, T-203 os-61, T-222, T-211, and T-204, respectively.

#### 2.2.2. Preparation of the Mechanical Pulp

As commented above, the initial biomass was classified to separate the stalks with diameters larger than 10 mm. These stalks were crushed and classified in an SM 2000 mill (Retsch, Haan, Germany). Then, the obtained biomass was submitted to a defibering process in a Sprout-Waldron equipment to obtain orange tree pruning mechanical pulp (OPMP). To increase the aspect ratio of the reinforcements, this process was run under cold aqueous conditions. The yield of the process measured as the ratio between the initial biomass and the obtained mechanical pulp was close to 100%.

#### 2.2.3. Compounding

The study includes two main steps where composite materials are prepared and characterized. In the first step, different percentages of coupling agent (MAPE), ranging from 0% to 10%, were added to a composite with a 20 wt.% OPFMP. These composites were prepared in a plastograph internal mixing machine (Brabender^®®^, Bremen, Germany). This machine can prepare lower amounts of composite materials and has a long processing time. The machine was operated at 180 °C for 10 min. The coupling agent was added together with the BioPE pellets. The obtained composites were pelletized with a knives mill and stored at 80 °C to eliminate any moisture.

In the second phase, the composites were mixed in a kinetic mixer (Gelimat^®®^, Ramsey, NJ, USA). The use of this equipment allows the production of higher amounts of composite in less time. The reduction in processing time is expected to provide reinforcements with higher aspect ratios since these reinforcements are submitted to lower attrition phenomena in the Gelimat^®®^ equipment than in the Brabender^®®^ mixer. BioPE, OPFMP, and coupling agent were introduced in the mixer at a 200 rpm. Then, the speed was increased to 3000 rpm for 2 min until a discharge temperature of 210 °C was reached. The obtained composites were pelletized with a knives mill and stored at 80 °C to eliminate any moisture.

#### 2.2.4. Mold Injection of the Standard Specimens and Mechanical Characterization

Composite pellets were dried at 80 °C for at least 24 h before mold injection. Then, dog bone standard specimens, in agreement with ASTM D638, were obtained by mold injection in a Meteor 40 device (Mateu & Sole, Barcelona, Spain). The temperatures of the three heating areas of the machine were 175, 180, and 190 °C, and the first and second pressures were 120 and 38 kg/cm^2^. At least 10 valid specimens were obtained for all the tested composites. Then, in agreement with ASTM D638, the obtained specimens were stored in a conditioning chamber for 48 h, at a 50% relative humidity and 23 °C. Composites were tensile tested in an 1122 Universal testing machine (Instron^TM^, Barcelona, Spain). The equipment was operated at 2 mm/min and was equipped with a 5 kN load cell. The mean tensile strength of the composites was computed form at least five valid tests.

#### 2.2.5. Recovering and Morphologic Analysis of the Fibers

The morphology of the reinforcements changes during the composite mixing and mold injection. Thus, to obtain the mean length and diameter of the reinforcements as well as the fiber length distributions, such reinforcement must be recovered from the composite. Thinning can be accomplished by matrix dissolution. In this case, the recovery was done on a Soxhlet equipment using decalin as the solvent. Small pieces of a standard specimen were placed into a cellulose filter, inside the Soxhlet equipment. The matrix was dissolved after a 24-h treatment. The recovered reinforcements were washed with acetone to eliminate all decalin and then rinsed with distilled water. The obtained fibers were measured in a MorFi Compact fiber analyzer (Techpap, Saint Martin d’Hères, France). Three samples were analyzed in each set.

## 3. Results and Discussion

### 3.1. Analysis of Chemical Composition

Table 1 shows the results obtained in the chemical characterization of orange tree prunings. They are compared to olive tree prunings and vine shoots (both seasonal agricultural residues, as in the case of orange tree prunings) and a widely used wood species such as eucalyptus [45,46,47,48].

The extractives contents are slightly higher than the values found in the literature for eucalyptus, being halfway between the values for olive tree prunings and vine shoots. The ash content, the highest value, can anticipate corrosion and clogging problems in the case of chemical processes to obtain cellulose fiber, which is not the case of the work since a mechanical treatment has been carried out. The lignin content has a very interesting value. It is similar to the values presented by agricultural residues and lower than the value of eucalyptus, and its interest lies in the capacity of lignin to favor links. Finally, regarding the holocellulose and α-cellulose content, it is important to highlight the good values they present, which are higher than those of the other agricultural residues. The presence of hydroxyl groups from holocellulose will undoubtedly favor the formation of links with MAPE, which will result in a better application of these renewable fibers of natural origin [46,47,48].

As can be seen in Table 2, the fibers obtained by mechanical treatment have a composition practically equivalent to the raw material (Table 1). These results are consistent with the treatment used which provides a yield close to 100% [49,50]. On the other hand, the chemical composition of the stone groundwood fibers shows how this treatment leads to an important removal of ashes and extractives which leads to a percentage increase in lignin content. On the other hand, the holocellulose content is also reduced, being 67%.

### 3.2. Study of the Effect of Coupling Agent Contents on the Tensile Properties of the Composites

Adding a natural fiber to a polymer can affect the tensile strength of a composite increasing or decreasing its value. In the first case, the fibers act as reinforcements, in the second case as fillers [51]. One of the main drawbacks of using lignocellulosic fibers as a polyolefin reinforcement is the hydrophilic nature of the surface of fibers. Polyolefin is hydrophobic, and, consequently, it is difficult to obtain a good fiber wetting when both phases are used without any treatment or the use of coupling agents. The literature shows that uncoupled composites usually show interfaces with noticeable voids that prevent transferring loads from the matrix to the fibers [52]. The use of coupling agents is one of the most successful strategies to increase interface strength and as result, the strength of the composites. Nonetheless, the percentages of coupling agents must be fine-tuned to avoid, on the one hand, the excessive use of reagents, and on the other hand unwanted effects due to an excessive percentage of such coupling agents. The literature shows how percentages in the range from 2 to 10 wt.% (regarding the fiber content), make possible increase the tensile strength of the composites. Further amounts of coupling agent tend to decrease slightly the tensile strength [50,52]. 

**Figure 1:**
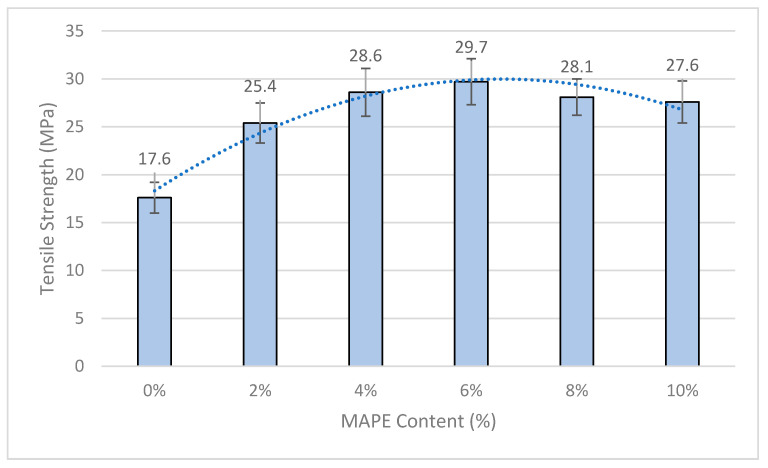
Evolution of the tensile strength of the composites reinforced with a 20 wt.% of OPFMP and different percentages of MAPE coupling agent.

Composite materials reinforced with a 20 wt.% of OPFMP and MAPE contents ranging from 0 to 10 wt.% were prepared and tensile tested. Figure 1 shows the evolution of the tensile strength of the composites against MAPE contents.

Uncoupled composites returned a tensile strength inferior to the BioPE matrix (18.05 MPa). In this case, OPFMP act as filler, and the absence of any interface between the fibers and the matrix hinders any potential strengthening of the fibers. Adding further percentages of MAPE increased noticeably the tensile strength of the composites. The authors found a local maximum value for the tensile strength of the composites when a 6 wt.% of MAPE was added to the mix. A higher coupling agent content produced a decrease in the tensile strength of the composites [53,54,55]. 

**Figure 2:**
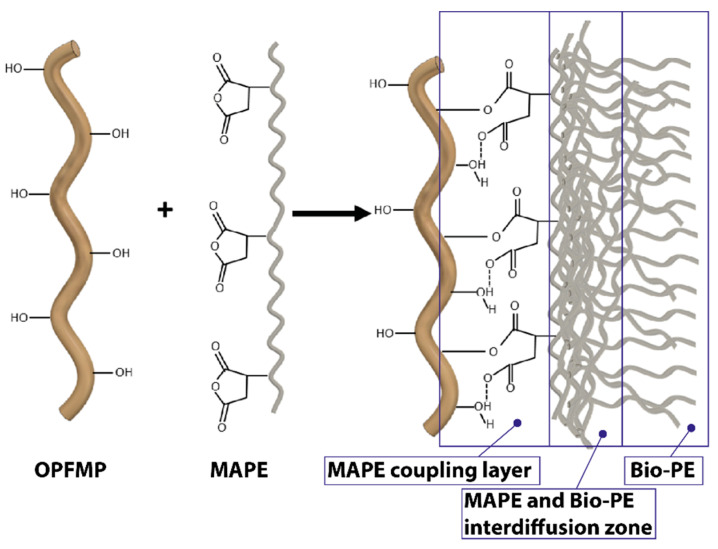
Interactions between the OPFMP and the coupling agent by the creation of covalent and hydrogen bonds, and entanglement of the MAPE PE chains with the Bio-PE matrix.

The use of maleic anhydride-modified polyethylene as a coupling agent is a rather attractive method because it avoids the use of expensive and toxic reagents [40]. The improvement of the interfacial adhesion between the fibers and the matrix leads to an improvement of the physical and mechanical properties of the biocomposite materials [39,56]. Figure 2 shows the interactions between OPFMP and the coupling agent as well as between MAPE and the matrix. The coupling agent (MAPE) is based on the polymeric matrix structure itself, chemically modified with functional groups capable of reacting with the hydroxyl groups of the fibers at the processing temperature. This agent has a double functionality, (i) the interaction with the polymeric matrix itself, through interdiffusion phenomena, due to its structural similarity with the polymeric matrix, and (ii) the capacity of reaction, at temperatures below 200 °C, of the maleic remains with the hydroxyl groups of the fiber, giving rise to a covalent fiber-matrix bond.

The percentage of MAPE agrees with the values found in literature and is the same that was found to ensure the highest tensile strengths when OPFMP was used as reinforcement for polypropylene (PP) [49]. 

The composites were prepared in a Brabender^®®^ mixer. This equipment is known to impact significantly on the morphology of the fibers, mainly on their length. This is due to the comparatively long processing times needed to prepare the composites. During these times the fibers are exposed to attrition phenomena that shorten the fibers [57].

### 3.3. Evaluation of the Tensile Properties of Coupled Composites

Composites with an OPFMP content in the range from 20 to 50 wt.% and 6% of MAPE coupling agent, were prepared as described in the materials and methods section to obtain standard specimens. In this case, the equipment used to prepare the composites was a Gelimat^®®^ mixer. This machine shortens the processing time and decreases the fiber shortening phenomena [57]. Table 3 shows the obtained results for the tensile properties.

The inclusion of OPFMP increased the tensile strength of the composites noticeably. The strength of the composites adding percentages of reinforcement ranging from 20 to 50 wt.% was, respectively, 60.1%, 79.4%, 102.6%, and 125.7% higher than the matrix. The composite at 20 wt.% prepared with the Gelimat^®®^ showed a slight increase in its tensile strength compared with the composite prepared with the Brebender^®®^ equipment (29.7 MPa).

The tensile strength of the BioPE is similar to a commercial high-density polyethylene (HDPE). For instance, a Rigidex HD5226EA (INEOS Polyolefin) HDPE reported 17.2 MPa tensile strength [58]. The same HDPE reinforced with a mechanical pulp from sugarcane bagasse returned 21.66 MPa tensile strength. In this case, the strength obtained with OPFMP is significantly higher. Nonetheless, the reinforcements came from two different sources and the chemical compositions of the surface of the fibers and the morphology of such reinforcements are different.

Other commodity composites are glass fiber (GF) reinforced polyolefin. These composites are among the most used in the industry due to its favorable relationship between cost and mechanical properties. However, the relationship between properties and the environmental impact of these composites is not as favorable. Thus, the industry is interested in finding more environmentally friendly alternatives. Glass fibers show a high intrinsic tensile strength and its presence in a composite ensures high increases of the tensile strength of such composites. The addition of 20% glass fiber to the HDPE matrix with 6% MAPE led to a tensile strength of 37.86 MPa, reaching 45.19 MPa when 30% of glass fiber was incorporated with 6% MAPE. In this sense, the lower intrinsic resistance of the fibers obtained means that the addition of 40% of OPFMP is necessary to reach the values of HDPE + 20%GF.

Polypropylene (PP) is another of the commodity matrices commonly used to obtain natural fiber-reinforced composites [59,60,61]. PP like Isplen PP099 K2M by Repsol-YPF (Tarragona, Spain), returned 28.4 MPa tensile strength. This strength is superior to BioPE’s [62,63]. Anyhow, a 20% OPFMP reinforced BioPE reaches similar tensile strengths easily. Thus, such composites can be proposed as alternatives to PP. The substitution has an environmental meaning, as the composite replaces an oil-derived polyolefin by a bio-based matrix and a natural fiber, both coming from renewable resources. Then, a 10% GF reinforced PP returns tensile strengths between 51 and 68 MPa [64]. From the data in Table 1, it can be seen that BioPE-based composites cannot compete with GF-reinforced polyolefin, in terms of tensile strength at break.

**Figure 3:**
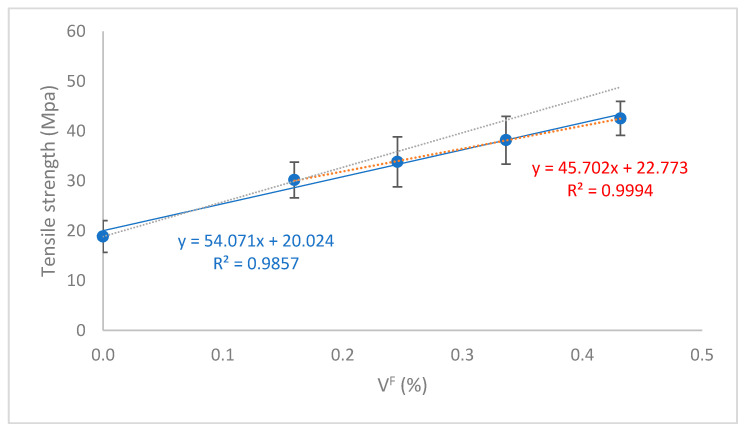
Evolution of the tensile strength of OPFMP reinforced BioPE composites against reinforcement volume fraction. The results have been fitted to a regression line.

The presence of the coupling agent allowed the transmission of loads from the matrix to the reinforcements. Some authors have pointed out a correlation between a linear increase of the tensile strength of the composites against their reinforcement volume fraction, and the existence of strong interfaces between the fiber and the polymer [65,66]. Moreover, a correlation between the linear evolution of the tensile strength and the good dispersion of the reinforcements has been detected by other authors [65,67]. Figure 3 shows the evolution of the tensile strength of the composites against fiber volume fraction.

The regression line obtained fits well with the experimental results. Only the composites with 20 wt.% of OPFMP (V^F^ = 0.160) deviated noticeably a regression line containing data on composites adding 20 to 50 wt.% OPFMP and BioPE. Nonetheless, the tensile strengths of the composites are almost perfectly aligned (red dot line). Based on the obtained results, the presence of an interface between the fibers and the matrix can be presumed. The maintained linear evolution of the tensile strength also allows us to assume a good dispersion of the fibers. The deviation of the matrix with a 20 wt.% OPFMP of the linear regression line can be due to a better dispersion of the fibers, as the lesser is its content the easier is obtaining a homogeneous dispersion without the creation of fiber bundles [68]. Another possible explanation can also be found in the presence of a stronger interface in the case of these composites. Finally, the morphology of the fibers may also affect the strengthening yield of OPFMP. The literature reports that the mean length of the reinforcement decreases when its percentage increases [69,70]. The morphology of the fibers, and especially its aspect ratio (ratio between the mean length and width of fiber) is known to affect the tensile strength of the composites. Thus, a study on the contribution of the fibers, the evolution of the morphology of the fibers, and the strength of the fiber-matrix interface will be needed to point out the effect of such parameters on the tensile strength of the composites.

The evolution of Young’s modulus against OPFMP content also shows the effect of such reinforcements on the stiffness of the composites. The increase in the modulus of the materials is remarkable and. compared with the matrix, the modulus of the composites with fiber percentages ranging from 20 to 50 wt.%, are 231%, 282%, 360%, and 444% higher, respectively. The same evolution has been observed for composites of HDPE reinforced with glass fiber, obtaining values of 3.1 and 4.4 GPa for composites with 20% and 30% GF respectively.

OPFMP composites show Young’s modulus higher than PP and its composites. BioPE composites showed higher Young’s modulus than PP-based materials reinforced with the same fibers [71]. At the same time, glass fiber reinforced composites show Young’s modulus of 4.6 and 6 GPa for materials at 20% and 30% GF content, respectively. Thus, BioPE composites reach Young’s modulus similar to a PP composite reinforced with 20% of GF [72]. The Young’s modulus is measured under short deformations. In these circumstances the loads transferred from the matrix to the reinforcement through the interface are low. Thus, a strong interface is not required. Consequently, the strength of the interface has a low impact on Young’s modulus of a short-fiber-reinforced composite. This fact has been widely reported in the literature [73,74]. On the other hand, literature correlates a linear evolution of Young’s modulus against the reinforcement volume fraction with good dispersion of the reinforcements.

In both cases, the tensile strength and Young’s modulus seems that the fiber loses some of its strengthening and stiffening capacity when its percentage is increased. It is possible to trace a line from the values of the matrix and the value of the composite at 20 wt.% of reinforcement content (blue line in Figure 3). The line shows the potential values of the tensile strength of the composites if the strengthening yield of OPFMP keeps constant. As was mentioned before, the causes can be related to the morphology of the reinforcements, its dispersion, and the strength of the interface.

### 3.4. Research on the Morphology of the Fibers

The initial geometry of the fibers, their initial mean length, and diameter, make sense in terms of processability. In this sense, long fibers or poorly individualized fibers are difficult to mix and mold. Thus, the fibers, previously to its mixing with the matrix, are cut down to processable length and individualized. The methods used to obtain a mechanical pulp ensure high yields (99%) regarding the initial raw material. Other processes allow obtaining fibers with surfaces capable of establishing higher chemical bonds than mechanical pulp, but these methods imply a lower yield. Thermomechanical or chemo-thermomechanical pulps ensure fiber surfaces with less lignin and extractives than mechanical pulp and, consequently higher amounts of cellulose and hemicelluloses. These last components are more reactive with the maleic phase of the coupling agents and ensure stronger interfaces. Nonetheless, the literature shows how, from a green chemistry point of view, mechanical pulps make more sense [75]. 

The literature agrees on the fact that composites with lower content of a reinforcement return higher average reinforcement lengths than composites with higher fiber contents. The cause of this fiber average length decrease is attributed to the fibers being exposed to attrition phenomena during the mixing operations [55]. To measure the mean length of the reinforcements, the fibers must be recovered from the composite and measured. Table 4 shows the mean geometric properties of the reinforcements against its content in the composite.

In the table, L_a_ refers to the mean length of the reinforcements recovered from the composites by matrix dissolution. Mean lengths reduce noticeably when it’s content in the composite increases. Compared with the composite with a 20 wt.% reinforcement content, the mean lengths of the composites at 30, 40, and 50 wt.% contents were 7%, 18%, and 26% shorter, respectively. 

**Figure 4:**
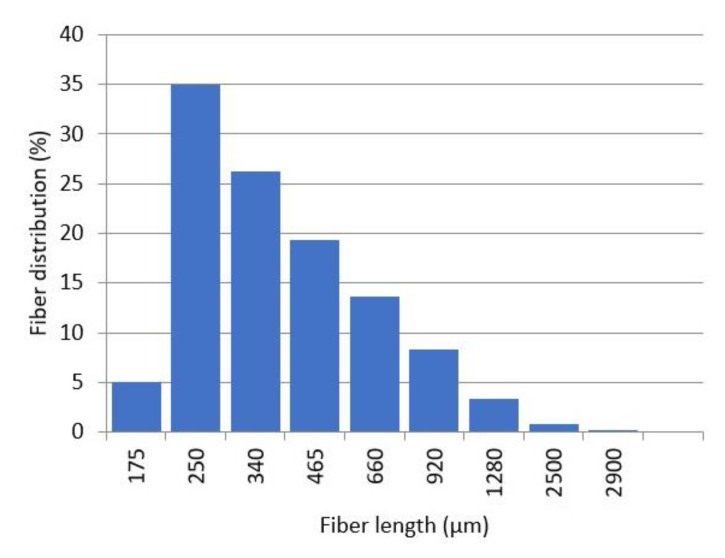
Fiber length distribution obtained after evaluating the OPFMP extracted from a composite at a 20 wt.% reinforcement content.

The mean diameter (D^F^) of the reinforcements suffered minor variations against the reinforcement content, and such variation had no statistical significance at a 95% confidence rate. Thus, the same mean diameter of 17.5 µm was considered for all the cases. The ratio between the mean length and diameter is known as aspect ratio, and the literature agrees on the fact that reinforcements with aspect ratios equal or higher than 10 can be considered fibers (in opposition to particles), and have strengthening and stiffening potential. All the reinforcements showed aspect ratios higher than 10. However, the aspect ratio of the reinforcements decreased with the reinforcement content, and this can be linked with a reduction of the strengthening potential of such reinforcements observed in Figure 3. Weighted lengths are used to prevent a misrepresentation of the longer fibers when micromechanics models are used to preview the contributions of the phases to a composite property. These models differentiate between subcritical and supercritical fibers. Supercritical fibers are those with a length that allows transferring a load equal to the intrinsic strength of the fiber. Then, supercritical fibers can contribute to a higher percentage than subcritical ones. Nonetheless, depending on the fiber length distribution the contribution of supercritical fibers can be underrated, and then weighted lengths are used. There are single (L_l_) and doubly (L_w_) weighted lengths, and depending on the shape of the distributions, one or the other will be used to obtain sensible results from the micromechanics models. Figure 4 shows the shape of the fiber length distribution of the fibers obtained from a composite with a 20 wt.% reinforcement content. The distribution shows how most of the fibers showed lengths below 1 mm. Thus, the use of a single or doubly weighted length can be previewed. The study of the evolution of the tensile strength of the composites against fiber content indicates a positive influence of such reinforcements. Nonetheless, knowing the neat contribution of such reinforcements can be of interest.

### 3.5. Neat Contributions of the Reinforcements to the Tensile Properties of the Composites

Micromechanics models are used to obtain the contributions of the phases of a composite to a property of such composite. The most used models are based on the rule of mixtures. The literature shows multiple versions for a rule of mixtures applicable to tensile properties of short fiber semi-aligned reinforced composites and commonly referred to as a modified rule of mixtures (mRoM) [75]. The following equation is mRoM for the tensile strength:

(1) $$\sigma_{t}^{C}=f_{c}\cdot \sigma_{t}^{F}\cdot V^{F}+\left(1-V^{F}\right)\cdot \sigma_{t}^{m*}$$

where $\sigma_{t}^{C}$ is the tensile strength of the composite. *V*^*F*^ is the reinforcement volume fraction. In equation, $\sigma_{t}^{F}$ is the intrinsic tensile strength of the reinforcement. The contribution of the fibers is equalized by a coupling factor (fc). The coupling factor has into account the effect of the fiber morphology, the strength of the interface, and the mean orientation of the fibers on the contribution of the reinforcements. $\sigma_{t}^{m^{*}}$ is the contributions of the matrix to the property. The contribution of the matrix to the tensile strength of the composite must be obtained from the experimental tensile test of the matrix. These contributions correspond to the tensile load states of the matrix at the breaking strains of the composite materials. To simplify obtaining these contributions, the stress-strain curve of the matrix was fitted to a 5th-grade polynomial equation:

(2) $$\sigma_{t}^{m*}=0.0006{\left(\varepsilon_{t}^{C}\right)}^{5}-0.01917{\left(\varepsilon_{t}^{C}\right)}^{4}+0.2618{\left(\varepsilon_{t}^{C}\right)}^{3}-1.8587{\left(\varepsilon_{t}^{C}\right)}^{2}+7.9779\varepsilon_{t}^{C}+0.2307$$

Equation (1) models the contribution of the reinforcements as its intrinsic property times a weighting factor times its volume fraction. All the parameters at the exception of the intrinsic properties and the weighting factors can be obtained from the tensile test of the matrix and the composites, and are shown in Table 3. Thus equation Equation (1) contains two unknowns and is unsolvable. Nonetheless, the net contribution of the fibers is the intrinsic property times the corresponding weighting factor. Rearranging Equation (1) it is possible to obtain the equation of a line as a function of the fiber volume fraction:

(3) $$f_{c}\cdot \sigma_{t}^{F}\cdot \left(V^{F}\right)=\sigma_{t}^{C}-\left(1-V^{F}\right)\cdot \sigma_{t}^{m*}=FTSF$$

**Figure 5:**
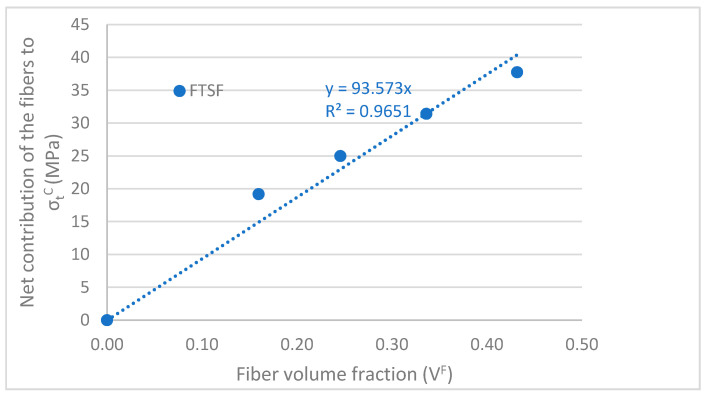
Net contribution of the reinforcements to the tensile properties of the composites: Fiber Tensile Strength Factor.

The contributions at the different volume fractions can be plotted, and the slope of the regression line will be a measure of the neat contribution of the reinforcements to the tensile property of the composite. The neat contribution to the tensile strength is referred to in the literature as Fiber Tensile Strength Factor (FTSF). Figure 5 shows the values obtained for the neat contributions to the tensile strength. The net contributions of the reinforcements allow comparing the yield of such materials as strengthening agents. Notwithstanding, knowing the intrinsic property and the weighting factor values can help in identifying the main factors impacting the direct contributions of the reinforcements.

### 3.6. Micromechanics of the Tensile Strength

Unlike the case of Young’s modulus, the strength of the interface has a great impact on the strengthening abilities of reinforcement [69]. Thus, the micromechanics of the tensile strength includes the strength of the interface between the fibers and the matrix as one of its main results. Besides, the models will permit knowing the theoretical intrinsic tensile strength of the reinforcements and an orientation factor that measures its mean orientation. To obtain such results the authors use a modified Kelly and Tyson model [76,77]. This model is a sophistication of a rule of mixtures and can be enunciated as:

(4) $$\sigma_{t}^{C}=\chi_{1}\left[X+Y\right]+Z$$

where *X*, *Y*, and *Z* are the contributions of subcritical fibers, supercritical fibers, and the matrix, respectively. The orientation factor χ_1_ weights the contribution of the reinforcements because they are not aligned with the loads. As it was mentioned before, the difference between subcritical and critical fibers is defined by the ability of the interface to transfer enough stress to reach the intrinsic tensile strength of the reinforcements. A strong interface will need less fiber length to transfer the same amount of stress than a weak interface. The critical length (*L_c_*) is defined by the following equation [78]:

(5) $$L_{c}=\frac{\sigma_{t}^{F}\cdot D^{F}}{2\cdot \tau }$$

In the equation, *τ* is the interfacial shear strength and accounts for the shear loads that the interface can transfer from the matrix to the reinforcement. Then, Equation (4) can be expressed as:

(6) $$\sigma_{t}^{C}=\chi_{1}\left(\overset{l=Lc}{\underset{l=0}{\sum }}\left[\frac{\tau \cdot l\cdot V_{l}^{F}}{D^{F}}\right]+\overset{\infty }{\underset{l=Lc}{\sum }}\left[\sigma_{t}^{F}\cdot V_{l}^{F}\cdot \left(1-\frac{\sigma_{t}^{F}\cdot D^{F}}{4\cdot \tau \cdot l}\right)\right]\right)+\left(1-V^{F}\right)\cdot \sigma_{t}^{m*}$$

Equation (6) has three unknowns; the interfacial shear strength, the intrinsic tensile strength, and the orientation factor. However, Bowyer and Bader proposed a numerical method that allows for solving the equation [79]. The method is based on supposing that all the phases are under the same strains and then the stress of the reinforcements at a defined strain can be expressed as this strain times the intrinsic Young’s modulus of the reinforcement. Then, by choosing two points on the elastic section of the composite stress-strain diagram, *τ* and *χ*_1_ can be obtained. Then, the remaining $\sigma_{t}^{F}$ can be obtained by solving Equation (6). Table 5 shows the parameters used to solve Equation (6) and obtain the micromechanics properties of the composites.

The authors used the doubly weighted length to solve the equation. Using arithmetical or single weighted lengths returned orientation factors that were considered erroneous. The two points chosen to apply Bowyer and Bader method were placed at 1/3 and 2/3 of the strain at the break of the composites. All the values for the contributions of the matrix were obtained by using Equation (2). The data for the composites were obtained from the experimental stress-strain curves of such composites. The contribution of the fibers was computed using the proposed strains and the mean intrinsic Young’s modulus of the reinforcements.

Table 6 shows the micromechanics properties obtained after solving Kelly and Tyson modified equation.

The orientation factor *χ*_1_ is related to the mean orientation of the semi-aligned fibers. In a mold injected piece there are different zones with different fiber mean orientation. The zone in contact with the mold tends to show fibers heavily oriented with the flow of the composite. On the other hand, the core zone tends to show fibers oriented perpendicular to the plastic flow. Thus, the mean orientation only informs on the fact that the fibers are not randomly oriented. The value of this parameter is linked to the equipment and the geometry of the injection mold, and previous experiences show that its expected values remain inside the range from 0.25 to 0.35 [65,80]. Thus, the orientation factor is used by the authors to assess the validity of the results and guide them on using single or doubly weighted lengths. All the composites returned a similar orientation factor. While the mean orientation of the fibers has a strong effect on the tensile strength of the composites and the higher is the alignment of the fibers with the loads, the stronger are the composite materials. 

The interfacial shear strength was in the range from 1084 to 9.18 MPa. Some authors use von Mises criteria to establish an upper bound for the value of the interfacial shear strength and a value to define a strong interface. Von Mises criteria are defined as a ratio between the tensile strength of the matrix and the square root of 3, in this case, 10.88 MPa. The obtained values are near to von Mises criteria and thus the composites show strong interfaces. 

The intrinsic tensile strength of the fibers showed values between 597 and 472 MPa. This value is in line with the literature, where the similar fibers as reinforcement for a polypropylene matrix showed an average intrinsic tensile strength of 512 MPa [52]. 

**Figure 6:**
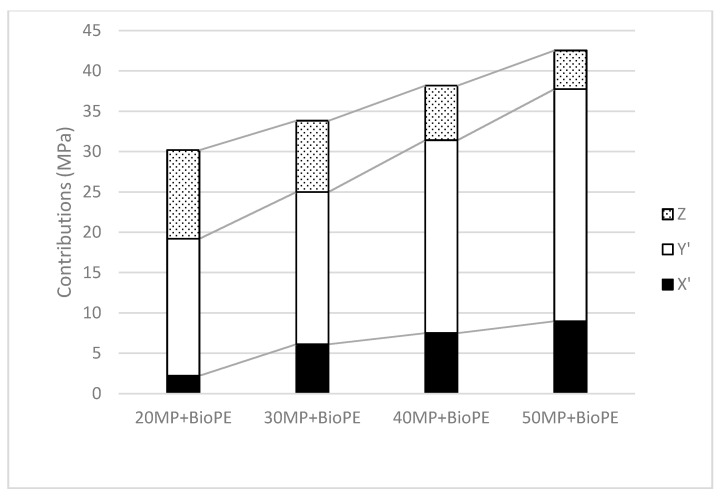
Contributions of the matrix, the subcritical, and the supercritical fibers to the tensile strength of the composites.

The value of the intrinsic strength decreases with the increasing percentages of reinforcement. This result agrees with the information shown in Figure 3, where the reinforcements showed decreasing yields in its strengthening abilities. Here again, the mean length of the fibers can be a major factor defining the strengthening ability of a fiber. Figure 6 shows the contribution of the phases to the tensile strength of the composites.

Supercritical fibers contribute always the most to the tensile strength of the composites, pointing out the importance of the mean length of the fibers. As expected, the contribution of the matrix decreases with the increasing percentages of fibers. This is due to two factors. On the one hand, the amount of matrix decreases, and its contribution does the same. On the other hand, the strain at break of the composites decreases, and the contribution of the matrix does also. Thus, the joint effect of these factors reduces the contribution of the matrix. The contribution of subcritical fibers increases with the amount of reinforcement, as expected due to the fiber’s shortening and the increasing amount of subcritical fibers.

## 4. Conclusions

Composite materials based on a bio-based polyethylene reinforced with mechanical pulp from orange tree prunings were mixed and tensile tested. Such composites included a 6% of coupling agent against reinforcement content. This coupling corresponded to the highest tensile strength results. Composites incorporated reinforcement contents in the range from 20 to 50 wt.%.

The composites showed an almost linear evolution of their tensile strength against reinforcement content. The deviation from a linear evolution was attributed to an increasing difficulty to disperse the fibers as its percentage increased. The composites showed tensile strength that allowed its use as polypropylene and some polypropylene composites substitute. Glass fiber reinforced polypropylene composites show tensile strengths higher than the obtained for BioPE based composites.

Young’s modulus of the composites showed a behavior against reinforcement content similar to its tensile strength. The Young’s modulus of the composites enabled them as PP and some of its composites substitutive, including GF reinforced PP up to 20 wt.% GF contents.

The contribution of the fibers to the tensile properties is noticeable, showing how it is possible to add value to such agroforestry waste. The use of coupling agents ensured the creation of strong interfaces between the fibers and the matrix.

The cost of BioPE compromises its competitiveness. Nonetheless, it is possible to substitute up to 50 wt.% of its content by a cheap filler/reinforcement like orange tree pruning fibers. Such fibers are agroforestry waste and are very cheap and can balance the costs of the final material. By using bio-based PE we avoid using oil-based polymers, and by using agroforestry waste as reinforcement we fulfill the principles of green chemistry and engineering and contribute to the circular economy.

## Figures and Tables

**Table 1:** Chemical compositions of raw material and different agriculture residues.

% *w/w*	Orange Tree Pruning	Olive Tree Pruning [43]	Vine Shoots [44]	Eucalyptus Wood [45]
Extractives	2.45	10.4	4.9	1.9
Ash	1.95	1.4	3.5	1.05
Lignin	20.8	19.7	20.3	24.1
Holocellulose	75.95	61.5	67.1	74.05
α-cellulose	49.05	35.7	41.1	-

**Table 2:** Chemical compositions of orange pruning mechanical pulp and stone groundwood.

% *w/w*	Orange Pruning Mechanical Pulp	Stone Ground Wood
Extractives	2.15	1.8
Ash	1.8	0.8
Lignin	20.7	29.9
Holocellulose	75.85	67.0

**Table 3:** Tensile properties of OPFMP reinforced BioPE composites and the BioPE matrix.

OPFMP % (% *w/w*)	*V^F^*	*σ_t_^C^* (MPa)	*E_t_^C^* (GPa)	*ε_t_^C^* (%)	*σ_t_^m^* * (MPa)
**0**	0	18.05	1.06	10.59	18.05
**20**	0.160	30.18	3.51	3.01	13.07
**30**	0.246	33.82	4.05	2.41	11.71
**40**	0.336	38.18	4.88	1.89	10.20
**50**	0.432	42.54	5.77	1.41	8.44

*V^F^* is the fiber volume fraction, $\sigma_{t}^{C}$ is the tensile strength, $E_{t}^{C}$ is the Young’s modulus and $\varepsilon_{t}^{C}$ is the strain at break of the materials, and *σ_t_^m^* * is the contribution of the matrix to the tensile strength of the composite.

**Table 4:** Average length of the OPFMP recovered from composites at different reinforcement contents. The table also shows the weighted mean lengths, the mean diameters, and the aspect ratio of the recovered reinforcements.

OPFMP % (% *w/w*)	L_a_ (µm)	L_l_ (µm)	L_w_ (µm)	D^F^ (µm)	L_a_/D^F^
**20**	359	498	695	17.5	20.5
**30**	332	460	643	17.5	19.0
**40**	292	405	566	17.5	16.7
**50**	266	368	514	17.5	15.2

**Table 5:** Parameters used to solve the Kelly and Tyson equation by using the method proposed by Bowyer and Bader.

Reinforcement Weight Content (%)	20	30	40	50
Average length (µm)	359.22	332.53	292.63	266.02
Weighted average length (µm)	695.16	643.51	566.29	514.81
Fiber modulus (GPa)	29.99	29.99	29.99	29.99
Elongation at break (%)	3.01	2.41	1.89	1.41
Strain level 1 analyzed (%)	0.99	0.80	0.62	0.47
Strain level 2 analyzed (%)	1.99	1.59	1.25	0.93
Composite strength (MPa)	30.18	33.82	38.18	42.54
Composite stress at strain level 1 (MPa)	17.50	19.00	19.40	18.30
Composite stress at strain level 2 (MPa)	28.00	30.50	32.00	31.50
Matrix stress at break (MPa)	13.07	11.71	10.20	8.44
Matrix stress at strain level 1 (MPa)	6.56	5.52	4.54	3.57
Matrix stress at strain level 2 (MPa)	10.51	9.15	7.75	6.24

**Table 6:** Micromechanics properties of the interface and the reinforcements of OPFMP reinforced BioPE composites.

OPFMP wt.%	20	30	40	50
χ_1_	0.305	0.307	0.308	0.308
τ (MPa)	10.84	9.18	9.29	9.53
L_c_ (µm)	482.17	508.23	473.31	432.89
σ_t_^F^ (MPa)	597.3	533.1	502.3	471.5
f_c_	0.20	0.19	0.19	0.19
